# Impaired deformability of circulating erythrocytes obtained from nondiabetic hypertensive patients: investigation by a nickel mesh filtration technique

**DOI:** 10.1186/s40885-015-0030-9

**Published:** 2015-10-07

**Authors:** Keita Odashiro, Kazuyuki Saito, Takeshi Arita, Toru Maruyama, Takehiko Fujino, Koichi Akashi

**Affiliations:** Department of Medicine, Kyushu University, Fukuoka, 812-8582 Japan; BOOCS Clinic, Fukuoka, 812-0025 Japan; Faculty of Art and Science, Kyushu University, Kasuga Kohen 6-1, Kasuga, 816-8580 Japan; Institute of Rheological Function of Foods Co., Ltd, Hisayama, 811-2501 Japan

**Keywords:** Deformability, Erythrocytes, Filtration, Hypertension

## Abstract

**Introduction:**

Hypertension is associated with microcirculatory disturbance, and erythrocyte deformability is a major determinant of the microcirculation. However, impairment of erythrocyte deformability in hypertensive patients in relation to antihypertensive treatment is unclear. The present study aimed to investigate this impairment in hypertensive patients under treatment using a highly sensitive and quantitative nickel mesh filtration technique.

**Methods:**

Deformability was evaluated by filterability, defined as the flow rate of a hematocrit-adjusted erythrocyte suspension relative to that of saline under a specific filtration pressure in a pressure-flow curve obtained by continuous filtration. Baseline characteristics of hypertensive patients (*n* = 101) and age-matched normotensive subjects (*n* = 14) were obtained from medical records, and diabetic patients were excluded.

**Results:**

Erythrocyte deformability in the hypertensive group was significantly (*p* = 0.010) lower (87.8 ± 2.2 %) than that of the normotensive group (89.4 ± 1.7 %) and inversely proportional (*r* = −0.303, *p* = 0.002) to the mean blood pressure (BP) measured on blood sampling for the filtration study. Stepwise multiple regression analysis demonstrated that this impairment was mostly attributable to the mean BP (*p* = 0.001), whereas current smoking and episodes of stroke or coronary artery disease were not contributors.

**Discussion:**

These findings indicate that erythrocyte deformability is impaired in the hypertensive patients, which depends on the current BP control rather than target organ damage.

## Introduction

Hypertension is a very common health-care problem in adult and elderly populations, and hypertensive complications such as stroke and coronary artery disease greatly increase medical expenditure [1]. Hypertension has rheologic impacts on circulating erythrocytes, and an abnormal erythrocyte rheology sustains a high blood pressure (BP) [2]. Oxidant stress accompanying this common disease disrupts the structure and function of oxygen-carrying human erythrocytes. Endothelial dysfunction in hypertensive patients also causes the mechanical and rheologic damage of erythrocytes. Although erythrocyte abnormalities in hypertensive patients have been widely reported [3, 4], studies focusing on erythrocyte deformability in relation to BP control are limited, i.e., optimal BP control from the viewpoint of erythrocyte rheology is a unique antihypertensive strategy to prevent target organ damage.

The deformability of erythrocytes passing through the microvascular network is an essential factor affecting microcirculation. However, the concept of erythrocyte deformability has not been strictly defined as a physical quantity, i.e., the evaluation of deformability depends on the relative sensitivity of the measurement technique. Since in vivo erythrocyte deformation involves bending, deformability is quantified by filterability, using a highly sensitive and quantitative nickel mesh filtration technique [5–7]. Therefore, we aimed to investigate the rheologic effects of hypertension on circulating erythrocyte deformability using this filtration technique.

## Methods

### Study population

This was a single-center, case-control study performed according to the Declaration of Helsinki (2008), i.e., signed informed consent was obtained from each subject prior to enrollment in the study. The study population consisted of 101 Japanese hypertensive patients and 14 age-matched normotensive subjects. All hypertensive patients were treated with antihypertensive drugs including Ca antagonists (65 %), angiotensin receptor blocking agents (40 %), angiotensin-converting enzyme inhibitors (17 %), and β-blocking agents (28 %), although combined antihypertensive drug prescription was common (36 %). These prescriptions were under the discretion of the treating physicians in outpatient clinics of the Fukuoka Seishukai Hospitals in the vicinity of Fukuoka City, Japan. Blood and urine examination, electrocardiography (ECG), echocardiography, and chest X-ray were routinely performed during regular monthly visits. Brain CT imaging and ambulatory ECG were conducted in 76 and 83 % of the hypertensive patients, respectively. Target organ damage in the hypertensive group was estimated by our colleagues in a blind manner. Lifestyle including smoking and drug prescription in each individual were evaluated by checking medical records. The normotensive group contained subjects visiting the outpatient clinic of this hospital once a year for health checkup. The body mass index (BMI) was calculated by the body weight (kg) divided by the square of the height (m^2^).

Atrial fibrillation (AF) was checked by standard and ambulatory ECG, and so was stroke by brain CT. Coronary artery disease (CAD) included a history of myocardial infarction, previous percutaneous coronary intervention (PCI), or coronary artery bypass grafting (CABG). The brachial BP was measured by the treating physicians on the morning of regular monthly visits, using a mercury sphygmomanometer in a sitting position after taking a few minutes rest. The systolic BP (SBP) and diastolic BP (DBP), measured on the day of blood sampling for erythrocyte deformability estimation, were used for statistical analysis. The mean BP was calculated as DBP + (SBP − DBP)/3. Exclusion criteria included diabetic patients showing HbA1c (National Glycohemoglobin Standardization Program (NGSP)) of more than 6.5 % or being prescribed antidiabetic agents, patients with hemodialysis and dementia, and those with prosthetic heart valve replacement. The study design was approved by the internal ethics committee of the Institute of Rheological Function of Foods, Co., Ltd. (Hisayama, Fukuoka, Japan).

### Erythrocyte suspensions

Erythrocyte suspensions were prepared as described elsewhere [8, 9]. About 10 mL of venous blood was sampled from the antecubital vein of subjects after overnight fasting using 21-gauge needles and disposable syringes (Terumo Japan, Tokyo, Japan) filled with 1/10 volume of 3.8 % trisodium citrate as an anticoagulant. Blood cell counting and hematocrit measurements were performed using a hemocytometer (Ace Counter, FLC-240A, Fukuda Denshi Co., Ltd., Tokyo, Japan). After centrifugation at 1300×*g* for 10 min, the supernatant was carefully aspirated to replace the buffy coat and plasma with saline buffered with N-(2-hydroxyethyl)-piperazine-N′-2-ethanesulfonic acid (HEPES) sodium salt (HEPES-Na). The composition of HEPES-Na-buffered saline (HBS) was NaCl 141 mM and HEPES-Na 10 mM. The osmolality and pH of the HBS were 287 mOsm/kg·H_2_O and 7.4, respectively. The osmolality of the HBS was measured using a freezing point depression-type osmometer (Fiske Mark 3 Osmometer, Fiske Associates, MA, USA). Intact erythrocytes were then washed three times by repeated resuspension with HBS and centrifugation at 800×*g*, 600×*g*, and 500×*g* for 10 min, respectively. The final hematocrit of the erythrocyte suspension was adjusted to 3.0 %. These procedures were performed within 2 h after blood sampling for a subsequent filtration study.

### Nickel mesh filter

**Fig. 1:**
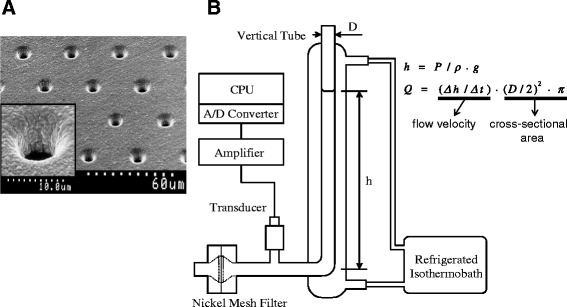
**a** Scanning electron microscopic photograph of a nickel mesh filter. Magnification of a single pore in the nickel mesh shows the smooth transition into the pore interior (inset). **b** Schematic illustration of the nickel mesh filtration system. The two equations indicate how to calculate the relationship between the flow rate (*Q*) and filtration pressure (*P*). The height of the meniscus (*h*) within the vertical tube was obtained by the continuous reduction of *P* during the filtration, specific gravity of the specimens within the tube (*ρ*), and acceleration of gravity (*g*). *Q* was calculated automatically by the first time derivative of *h* (dh/dt) and internal cross-sectional area of the tube (*D*, internal diameter of the vertical tube). The obtained *P*-*Q* relationship is displayed on the computer screen

Figure 1a shows an electron microscopic photograph of a nickel mesh filter that was produced in accordance with our specifications by a photofabrication technique (Dainippon Printing Co., Ltd., Tokyo, Japan). We specified that this filter should have an outer diameter of 13 mm, have a filtration area of 8 mm in diameter, be 11-μm thick, and have an interpore distance of 35 μm (Tsukasa Sokken Co., Ltd., Tokyo, Japan). The vertical and cylindrical pores were distributed regularly across the filter without coincidence or branching. The pore entrances exhibited round and smooth transition into the pore interior. Pore diameters are all exactly identical in a specific nickel mesh filter. Filters with a specific pore diameter ranging from 3.0 to 6.0 μm are available for selection depending on the suspension materials. After repeated preliminary experiments to choose an appropriate pore size, a nickel mesh filter with a pore diameter of 4.94 μm was used.

### Erythrocyte filterability

A filtration study was performed blindly using a nickel mesh filtration apparatus (Model NOBU-II, Tsukasa Sokken Co., Ltd., Tokyo, Japan), as shown in Fig. 1b. In brief, the relation between hydrostatic pressure (*P*; mmH_2_O) and time (*t*; second) was obtained during continuous filtration using a pressure transducer. *P* was transformed to the height of a meniscus in a vertical tube (*h*; mm). The tangent of the *h*-*t* curve determined by drawing points corresponding to different heights gives the rate of fall of the meniscus (dh/dt). Thereafter, by multiplying the rate of fall by the internal cross-sectional area of the vertical tube, the relation of flow rates (*Q*; mL/min) and corresponding *P* (*P*-*Q* curve) was obtained, which is a basic of hemodynamics [10, 11]. This procedure was automatically performed by measurement software installed on a personal computer (DELL Latitude CS, Dell Inc., Round Rock, TX, USA) and monitored on the main window of the computer screen. Together with the start of data acquisition, the measurement software displays the *h*-*t* curve continuously during the filtration process. When the filtration has been completed, the software displays the *P*-*Q* curve. The *h*-*t* and *P*-*Q* curves are stored simultaneously in Microsoft Office Excel 2003 on Windows XP (Microsoft, Tokyo, Japan). The temperature of the specimens was kept at 25 °C by circulating isothermal water through a water jacket surrounding the vertical tube (Fig. 1b). The flow rate of the erythrocyte suspension as a percentage of that of HBS at 100 mmH_2_O was used as an index of erythrocyte deformability. These experiments were performed at room temperature (22 ± 3 °C).

### Erythrocyte shape

An aliquot of the erythrocyte suspension was fixed with an isotonic 1.0 % glutaraldehyde solution containing 24.5 mM NaCl and 50 mM phosphate buffer (pH 7.4). The shape of erythrocytes was observed blindly by collaborators using a differential interference contrast microscope (Diaphoto 300, Nikon Co., Ltd., Tokyo, Japan) at ×400 magnification.

### Data analyses

All data are expressed as means ± SD. For statistical analyses, a sample size was chosen that provides 90 % power with an *α* error of 0.05 based on our previous study [12], being ≥82 cases. The Kolmogorov-Smirnov test was used for normality. Comparison of normally distributed continuous variables between the two groups was conducted with the unpaired Student’s *t* test, and that of other variables was performed by the Mann-Whitney *U* test. Stepwise multiple regression analysis was used to determine the significant contributors to erythrocyte deformability impairment. None of the variables with missing data qualified. The criterion for entering into the regression model was a significant correlation coefficient or otherwise clinically meaningful variables. Multi-collinearity was considered to be a variance inflation factor of more than 10. Multiple comparison among tertile subgroups in the hypertensive patients was performed using the Kruskal-Wallis test. These analyses were performed using PASW software (Windows version 18.0; SPSS, Chicago, IL, USA). Differences with *p* < 0.05 were considered significant.

## Results

### Patient profiles

Baseline characteristics of the hypertensive and age-matched normotensive groups are detailed in Table 1. Silent stroke (lacunar infarction) was found in 12 patients, and 15 patients with CAD were included in the hypertensive group. The prevalence of obesity, defined as BMI of more than 25 kg/m^2^ (32.7 vs. 28.6 %, *p* = 0.704), and that of current smokers (*p* = 0.754) were equivalent between the two groups. No significant differences in HbA1c (NGSP) or the serum lipid profile were found between the two groups. Hematology showed equivalent erythrocyte properties.

**Table 1:** Baseline characteristics of the hypertensive and normotensive groups

	Hypertensive group (*n* = 101)	Normotensive group (*n* = 14)	*p* value
Age (years)	66.6 ± 10.7	64.2 ± 4.4	0.421
Gender (female/male)	41 / 60	6 / 8	0.872
BMI (kg/m^2^)	23.5 ± 3.0	22.6 ± 3.4	0.258
Current smoker (%)	29 (28.7)	3 (21.4)	0.754
HbA1c (NGSP) (%)	5.2 ± 0.5	5.2 ± 0.3	0.547
Total cholesterol (mg/dL)	203.2 ± 36.8	202.1 ± 35.5	0.918
HDL cholesterol (mg/dL)	58.2 ± 15.1	62.0 ± 11.5	0.364
LDL cholesterol (mg/dL)	111.1 ± 29.6	125.1 ± 34.3	0.105
Triglyceride (mg/dL)	139.7 ± 100.7	110.7 ± 72.0	0.301
Systolic BP (mmHg)	143.2 ± 18.9	125.1 ± 15.5	<0.001
Diastolic BP (mmHg)	81.0 ± 11.3	72.0 ± 3.3	0.004
Hb (g/dL)	14.0 ± 1.8	13.4 ± 1.1	0.189
Ht (%)	40.4 ± 4.5	38.7 ± 2.9	0.184
MCV (fl)	93.8 ± 4.7	91.7 ± 3.8	0.110
MCH (pg)	32.6 ± 1.9	34.6 ± 0.8	0.651
MCHC (g/dL)	34.7 ± 1.0	31.7 ± 1.5	0.109
Erythrocyte filterability (%)	87.8 ± 2.2	89.4 ± 1.7	0.010

*BMI* body mass index calculated by body weight (kg) divided by square of height (m^2^), *BP* blood pressure, *Hb* hemoglobin, *HbA1c(NGSP)* hemoglobin A1c concentration estimated according to National Glycohemoglobin Standardization Program, *MCHC* mean corpuscular hemoglobin concentration, *MCV* mean corpuscular volume

### Erythrocyte filterability

**Fig. 2:**
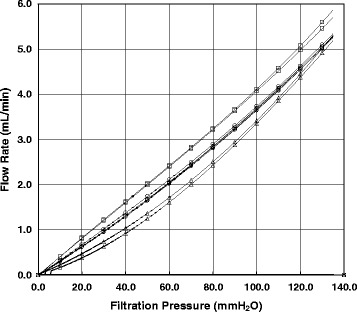
Representative relationships between filtration pressure (*P*; mmH_2_O) and flow rate (*Q*; mL/min) during the continuous filtration experiment using erythrocyte suspensions and HEPES-buffered saline. The *P*-*Q* relationships correspond to saline passages (*empty square*), filtrations of erythrocyte suspension obtained from the hypertensive patients (*empty triangle*), and those of the age-matched normotensive subjects (*empty circle*), respectively. *P*-*Q* curves in control saline were completely superimposable, indicating reproducibility. Flow rates in the hypertensive group were lower than those in the normotensive group at any given filtration pressure

Figure 2 shows representative *P*-*Q* curves for saline and erythrocyte suspensions obtained from the hypertensive and normotensive patients under the continuous filtration process. Saline behaved as Newtonian fluid, showing a linear *P*-*Q* relationship passing through the origin. The linearity was superimposable, indicating the excellent reproducibility of this filtration system. In contrast, the *P*-*Q* relationships for the erythrocyte suspensions displayed smooth convex curves along the abscissa over the low-pressure region, showing non-Newtonian behaviors. This trend was evident in the hypertensive group compared with the normotensive group, i.e., the flow rate of the erythrocyte suspension of the hypertensive patients was always lower than that of the normotensive subjects at any given filtration pressure.

### Correlation between BP and filterability

**Fig. 3:**
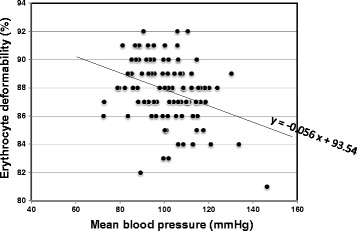
Individual erythrocyte deformability (%) is plotted as a function of the corresponding mean blood pressure (BP; mmHg) in the hypertensive patients (*n* = 101). Erythrocyte deformability is inversely proportional to the mean BP (*r* = −0.303, *p* = 0.002), showing a regression line of *y* = −0.056*x* + 93.54

The mean erythrocyte deformability in the hypertensive patients (87.8 ± 2.2 %, *n* = 101) was slightly but significantly (*p* = 0.010) impaired relative to that in the age-matched normotensive subjects (89.4 ± 1.7 %, *n* = 14, Table 1). Table 2 summarizes the correlation between the erythrocyte deformability and demographic variables in the hypertensive patients. The listed variables include covariates of hypertension or contributors to erythrocyte deformability in our recent studies [8, 9, 12]. The associations of erythrocyte deformability with the mean BP alone was significant (*r* = −0.303, *p* = 0.002), yielding the regression line of deformability as a function of the mean BP, as in *y* = −0.056*x* + 93.54 (Fig. 3). The difference of deformability with respect to AF did not reach significance (*p* = 0.060).

**Table 2:** Correlation of erythrocyte filterability and demographic variables in the hypertensive patients

Continuous parameters	Correlation coefficient	*p* value
BMI (kg/m^2^)	0.161	0.109
HbA1c (NGSP) (%)	0.140	0.163
Total cholesterol (mg/dL)	0.076	0.450
Mean BP (mmHg)	−0.303	0.002
Discrete parameters	*n*	*p* value
Atrial fibrillation	11	0.060
Current smoker	29	0.864
Stroke episode	12	0.211
Coronary artery disease	15	0.821

Abbreviations are shown in Table 1

Since hypertension is associated with various covariates, stepwise multiple regression analysis was performed to identify significant contributors to impaired erythrocyte deformability. A clinically relevant regression model including covariates of hypertension showed a significant multiple correlation coefficient (*R* = 0.327, *p* = 0.001). In this model, the mean BP (*p* = 0.001) alone was a significant variable contributing independently to the impaired deformability (Table 3). Although BMI was positively correlated with serum total cholesterol (*r* = 0.223, *p* = 0.025) in the hypertensive patients, multi-collinearity was unlikely considering the variance inflation factors (≈1.0). The mean erythrocyte deformability was compared among the tertiles of the hypertensive groups divided by the BP control state. The difference in erythrocyte deformability was significant (*p* = 0.009), i.e., the deformability was higher according to the tertiles showing lower SBP and DBP (Table 4). The deformability of the tertile showing the lowest BP in the hypertensive patients (88.6 ± 2.1 %) was not different from that of the normotensive subjects (89.4 ± 1.7 %, *p* = 0.185). Moreover, BP in this tertile was equivalent to that in the normotensive group (*p* = 0.467 in SBP, and *p* = 0.173 in DBP).

**Table 3:** Multiple regression analysis predicting contributors to erythrocyte filterability

Covariate	Variance inflation factor	*t* value	*p* value
BMI (kg/m^2^)	1.001	1.503	0.136
HbA1c (NGSP) (%)	1.017	0.401	0.689
Total cholesterol (mg/dL)	1.006	1.499	0.137
Mean BP (mmHg)	1.000	−3.431	0.001
Atrial fibrillation	1.044	1.143	0.256
Stroke episode	1.004	−1.498	0.137

Abbreviations are shown in Table 1

**Table 4:** Tertile comparison of erythrocyte filterability among the hypertensive patients

Tertile	Systolic BP	Diastolic BP	Erythrocyte filterability	*p* value
(*n*)	(mmHg)	(mmHg)	(%)	
First (34)	160.7 ± 12.3	92.9 ± 6.9	87.0 ± 2.1	
Second (34)	145.3 ± 9.4	79.7 ± 6.3	87.9 ± 2.3	0.009
Third (33)	122.4 ± 9.6	69.8 ± 5.5	88.6 ± 2.1	

### Erythrocyte shape

Morphologically, erythrocytes obtained from the hypertensive and normotensive groups did not show any discernible shape changes.

## Discussion

### Main findings

The main findings of this study using the sensitive, reproducible, and quantitative nickel mesh filtration technique (Fig. 1) are that erythrocyte deformability was impaired in patients with hypertension even under treatment (Table 1) and that this impairment was inversely proportional to the mean BP (Table 2). Because a temporary high BP alone was a significant contributor to the corresponding erythrocyte deformability (Table 3), this study indicates that intensive BP control should be recommended to resolve the impaired erythrocyte deformability and microcirculatory disturbance in the hypertensive patients (Table 4). Multiple regression analysis was able to determine contributors to the impaired erythrocyte deformability when this rheologic parameter was obtained with a highly sensitive and reproducible filtration technique.

### Hypertension and hemorheology

Clinical hypertension usually contains covariates such as obesity, diabetes, and dyslipidemia, which may influence intact erythrocyte deformability. Actually, our previous studies using the same technique as in this study demonstrated that these covariates have a potential impact on deformability [8, 9, 12]. Therefore, the present study excluded diabetic patients. The serum lipid profile in the hypertensive group did not differ from that of the normotensive group, and BMI in the two groups was equivalent (Table 1).

Intact erythrocyte deformability in the hypertensive group (87.8 ± 2.2 %, *n* = 101) was slightly but significantly (*p* = 0.010) reduced compared with that in the age-matched normotensive group (89.4 ± 1.7 %, *n* = 14, Table 1). The difference in the mean erythrocyte deformability between the two groups may be small (Fig. 2). However, flow-pressure curves of control saline were superimposable (100 % as a positive control), and the deformability of human erythrocytes subjected to the acute oxidant stress in vitro was impaired to nearly 0 % (negative control), which means that the nickel mesh pores were completely clogged by oxidatively damaged erythrocytes [13]. Considering these positive and negative controls, it is conceivable that the level of impaired erythrocyte deformability observed in this study may have a potential rheologic impact on the microcirculation in vivo.

It is generally accepted that erythrocyte deformability is mainly determined by (1) the erythrocyte membrane structure and properties, (2) the internal viscosity that is reflected by mean corpuscular hemoglobin concentration MCHC, and (3) the cellular size and other geometric factors reflected by mean corpuscular volume (MCV) and the erythrocyte shape [6, 10, 11, 14]. Therefore, abnormal membrane properties, increases in MCHC or MCV, and several kinds of shape change impair the erythrocyte deformability individually or in concert. Considering that there were no discernible shape changes and there were equivalent MCV and MCHC in the two groups (Table 1), the impairment of erythrocyte deformability in the hypertensive group mainly arises from erythrocyte membrane properties.

Abnormal erythrocyte membrane properties and functions have been reported by extensive laboratory investigations [2–4]. Although further studies are required concerning whether these membrane abnormalities are genetic or environmental in hypertensive patients, it is likely that they are based mostly on the oxidative stress caused by the systemic or local production of reactive oxygen species (ROS) reported in experimental and clinical hypertension [15, 16]. Human erythrocytes are so sensitive to oxidative stress [17, 18] that hypertensive erythrocytes demonstrate membrane lipid peroxidation and reduced antioxidative enzyme activities [4]. An impaired redox balance of hypertensive erythrocytes reduces the membrane fluidity which is closely associated not only with deformability but also with transmembrane ion transport, cytosolic Ca^2+^ handling, and internal pH regulation [19].

Hypertensive erythrocyte membrane abnormalities are also based on the mechanical stress accompanying vasoconstriction derived from vascular endothelial dysfunction [20]. In our recent study of spontaneously hypertensive rats (SHR), impaired erythrocyte deformability was marked in the pre-hypertensive stage relative to the established hypertensive stage, suggesting that this rheologic abnormality may be a prerequisite of hypertension development [21]. Impaired erythrocyte passage in the microvasculature causes mechanical damage to the stagnant erythrocyte membrane, insufficient oxygen delivery, and tissue hypoxia, leading to further vasoconstriction and BP elevation [2]. This vicious rheologic cycle underlies hypertension development. The human erythrocyte lifespan is about 120 days, and this filtration technique confirmed that the mechanical impairment of human erythrocyte deformability in vitro is reversible [7]. Therefore, the present study revealed that the deformability is mostly linked to temporary BP elevation (Table 3), and the fact that inadequate BP control cannot reverse the impaired deformability (Table 4) is compatible with this vicious rheologic cycle theory.

### Clinical perspectives

There is a large body of evidence that strict BP control is required in the hypertensive patients to prevent target organ damage, whereas evidence that impaired erythrocyte deformability is associated with poor outcome in the hypertensive patients is scanty. Fornal et al. reported the significant correlation between the erythrocyte deformability and the left ventricular (LV) geometry calculated by LV mass index, i.e., showing that LV mass index increases with impaired deformability which elevates arterial resistance and cardiac afterload [22]. Considering that LV hypertrophy (LVH) is a predictor of cardiac mortality and morbidity, rheologic abnormalities has potential impact on outcome in the hypertensive patients.

In the Candesartan Antihypertensive Survival Evaluation in Japan (CASE-J) trial, the risk of cardiac events in the hypertensive patients showing LVH was reduced and equivalent to that of those without LVH when strict BP control (SBP less than 130 mmHg and DBP ranging from 75 to 79 mmHg) was achieved [23]. Target organ damage in the hypertensive patients per se is promoted by oxidant stress and procoagulant, proinflammatory effects mediated partly by the angiotensin type 1 (AT1) receptor via the toll-like receptor (TLR) [24]. These harmful effects cause marked erythrocyte membrane damage, and impaired microcirculation accelerates hypertensive complications. Actually, strict (SBP less than 130 mmHg) and not lenient (SBP less than 140 mmHg) BP control has been reported to suppress the innate immune response, leading to an inflammatory process via TLR of peripheral monocytes in the nondiabetic hypertensive subjects, as in this study [25]. The results of our study are consistent with these findings in terms of strict BP control resolving the impaired erythrocyte deformability and microvascular obstruction in the nondiabetic hypertensive patients.

### Limitations

This study has a few limitations. This single-center, cross-sectional study clarified the BP-dependent alteration of erythrocyte deformability in the hypertensive patients (Table 4). However, a longitudinal study is important to confirm this phenomenon. Moreover, antihypertensive treatment depended on the discretion of the treating physicians and was not randomized. Individual antihypertensive drug’s effects on the deformability were not investigated, because combined antihypertensive drug prescription was common. Considering that temporary BP elevation alone was a contributor to the deformability (Table 3), vascular tonus regulating flow resistance rather than neuro-humoral factors modified by antihypertensive drugs may have played a major role in the erythrocyte rheology.

## Conclusion

The present study, using a nickel mesh filtration technique, clarified that erythrocyte deformability based on the pressure-flow curve in a filtration study is significantly impaired in the nondiabetic hypertensive patients even under treatment. Multiple regression analysis demonstrated that the level of this impairment depends on temporary BP control and not on the history of hypertensive complications such as stroke or myocardial infarction. Although a large cohort is required, this study suggests that optimal antihypertensive treatment is required to maintain circulating erythrocyte deformability, facilitating efficient microcirculation.

## Abbreviations

AF: atrial fibrillation
BMI: body mass index
BP: blood pressure
CABG: coronary artery bypass grafting
CAD: coronary artery disease
CASE-J: Candesartan Antihypertensive Survival Evaluation in Japan
DBP: diastolic blood pressure
HBS: HEPES-Na-buffered saline
HEPES: N-(2-hydroxyethyl)-piperazine-N′-2-ethanesulfonic acid
LVH: left ventricular hypertrophy
MCH: mean corpuscular hemoglobin
MCHC: mean corpuscular hemoglobin concentration
MCV: mean corpuscular volume
PCI: percutaneous coronary intervention
ROS: reactive oxygen species
SBP: systolic blood pressure
SHR: spontaneously hypertensive rats
TLR: toll-like receptor
